# Interaction network among functional drug groups

**DOI:** 10.1186/1752-0509-7-S3-S4

**Published:** 2013-10-16

**Authors:** Minho Lee, Keunwan Park, Dongsup Kim

**Affiliations:** 1 Department of Bio and Brain Engineering, Korea Advanced Institute of Science and Technology, 291 Daehak-ro, Yuseong-gu, Daejeon, 305-701 Korea

## Abstract

**Background:**

More attention has been being paid to combinatorial effects of drugs to treat complex diseases or to avoid adverse combinations of drug cocktail. Although drug interaction information has been increasingly accumulated, a novel approach like network-based method is needed to analyse that information systematically and intuitively

**Results:**

Beyond focussing on drug-drug interactions, we examined interactions between functional drug groups. In this work, functional drug groups were defined based on the Anatomical Therapeutic Chemical (ATC) Classification System. We defined criteria whether two functional drug groups are related. Then we constructed the interaction network of drug groups. The resulting network provides intuitive interpretations. We further constructed another network based on interaction sharing ratio of the first network. Subsequent analysis of the networks showed that some features of drugs can be well described by this kind of interaction even for the case of structurally dissimilar drugs.

**Conclusion:**

Our networks in this work provide intuitive insights into interactions among drug groups rather than those among single drugs. In addition, information on these interactions can be used as a useful source to describe mechanisms and features of drugs.

## Background

Recently, various drug-related properties including therapeutic target, off-target, activity, toxicity, pharmacokinetic properties and side effect have been successfully described by some elegant methods such as analysis of chemical structures [1], docking simulation [2-4], chemical-genetic profile [5-7], connectivity map [8], target-sharing information, and side-effect similarity [9]. To go further and to treat complex disease like cancer, these recent advances in drug discovery make people pay more attention to a novel approach named polyphamacology [10] than to the design of a particular drug which could target disease-causing genes, because most of complex diseases result from vast range of biological abnormalities. This attention to polypharmacology also involves increasing attention to detect rational combination of older drugs to treat novel diseases. In this paradigm, significant attention to drug-drug interaction (DDI) is necessarily critical to detect ideal drug combinations to have synergistic clinical effects [11].

Another field, where DDI information would be significant, is combinatorial drug toxicology [12]. In some cases, DDIs exhibit adverse drug reactions (ADRs) [13] or critical threat to patients with multiple medications [14-16]. For example, about 4% of causes of death of cancer patients were originated from DDIs [17]. The risk of toxicity caused by unwanted polypharmacology is emerging threat to public health [18]. Thus, it is quite important to detect and predict DDIs in both drug discovery and drug toxicology.

It is essential to use system-based approach, or network-based in order to detect beneficial and harmful drug combination, because DDI necessarily involves the complexity. Many recent approaches regarding DDI have utilized information from networks among human diseases, biology, and chemistry [19,20] such methods as prediction of DDIs through protein-protein interaction (PPI) network [21], prediction of ADRs by integrating PPI network and drug structures [22] and a method using drug-target interaction network [23]. There have been also some researches on DDI using network analysis. Hu et al. constructed DDI network of 966 drugs and simply reported some properties of their DDI networks [24]. More advanced work by Xu et al. (drug cocktail network) developed drug combination predictor based on DDI network [25]. However, analysis of systematic DDI relationship in terms of functional context has not been made so far.

In this study, we perform systematic analysis of DDIs based on functional group of drugs and construct the network which consists of interactions among the drug groups (DGs). The classification we used are based on the anatomical therapeutic chemical (ATC) classification system [26]. The resulting DG-DG interaction network provides more contextual and intuitive view on DDI. In addition, the secondary network, whose edge represents that two DGs share a number of DG-DG interactions, is also constructed to detect a set of DGs showing similar DG-DG interaction patterns. We show that the DGs that have similar interactions share many similar drug features, suggesting that DDIs contain the information about drug mechanisms. Moreover, we question whether DDI information can be used to infer drug mechanisms by investigating common drug features for interacting drug pairs. The results show that some drug features such as metabolizing enzyme, drug function, and target pathway are well-described by DDI even for the case that chemical structure similarity is low.

## Results and discussion

### Statistically significant DG-DG interactions

**Figure 1:**
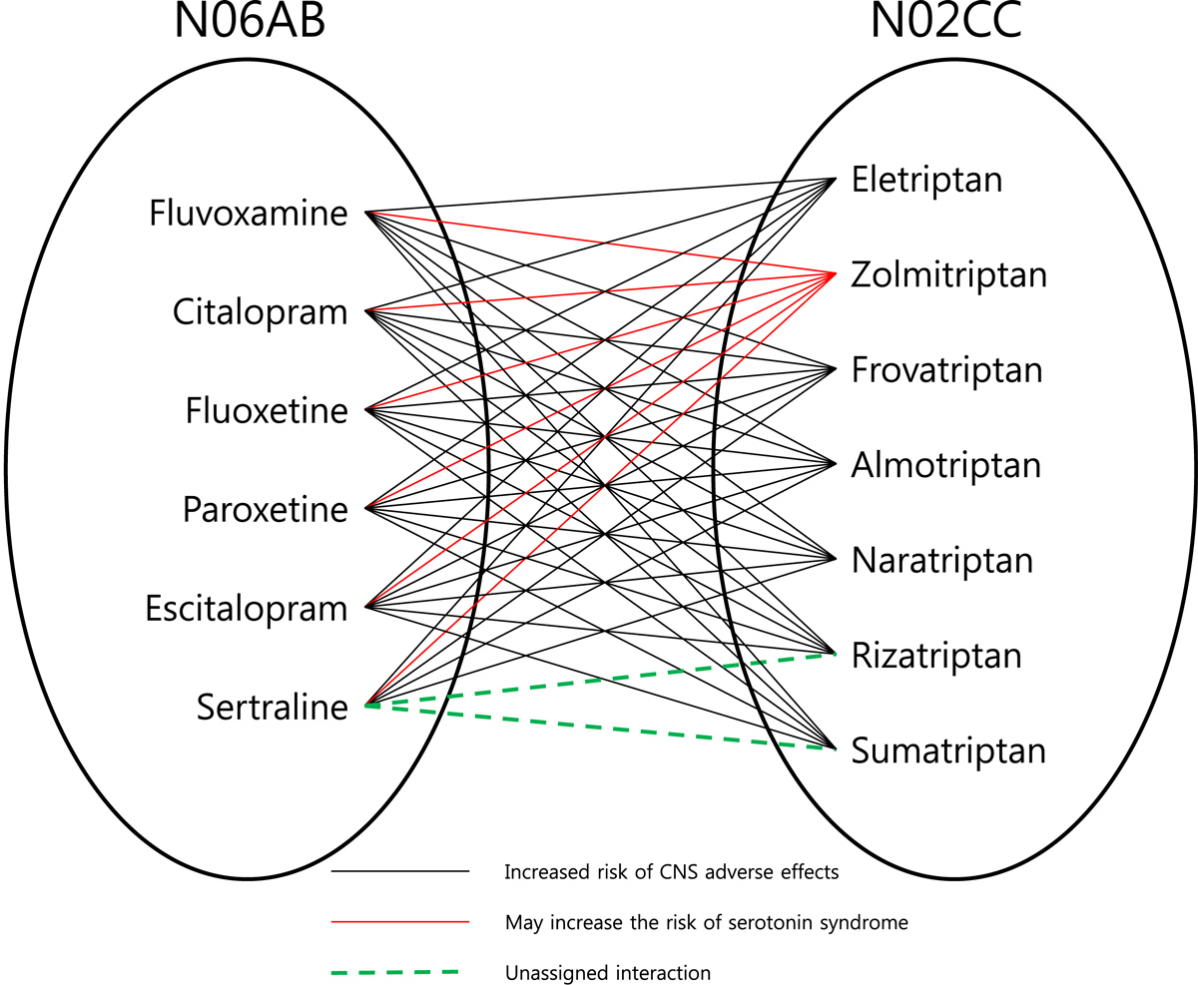
**Example of DG interactions, and prediction of new DDI**.

For a pair of functional DGs where there are significantly large numbers of drug interactions, it is reasonable that the DGs are functionally related and the drugs composing those two are highly likely to have drug interactions even for the drug pairs whose interaction was not assigned or not identified (see Methods). For example, N06AB (Selective serotonin reuptake inhibitors; six members in DrugBank) and N02CC (Selective serotonin (5-HT1) agonists; seven members in DrugBank) have forty assigned drug interactions in DrugBank, except two pairs out of forty-two possible drug pairs (Figure 1). The two drug-groups commonly refer serotonin-involved groups and most interaction types are the same, "increased risk of CNS adverse effects", except for the interactions of Zolmitriptan. Therefore, we expected that the not annotated two pairs also would have similar drug interactions (dotted line in Figure 1), and fortunately, those interactions were annotated in drugs.com with the "major interaction" class, representing high clinical significance.

Like this example, our analysis on drug-group interactions can provide the information about not only the meaningful drug-group interactions but also missing drug-drug interactions.

### Systematic interaction map among functional DGs

**Figure 2:**
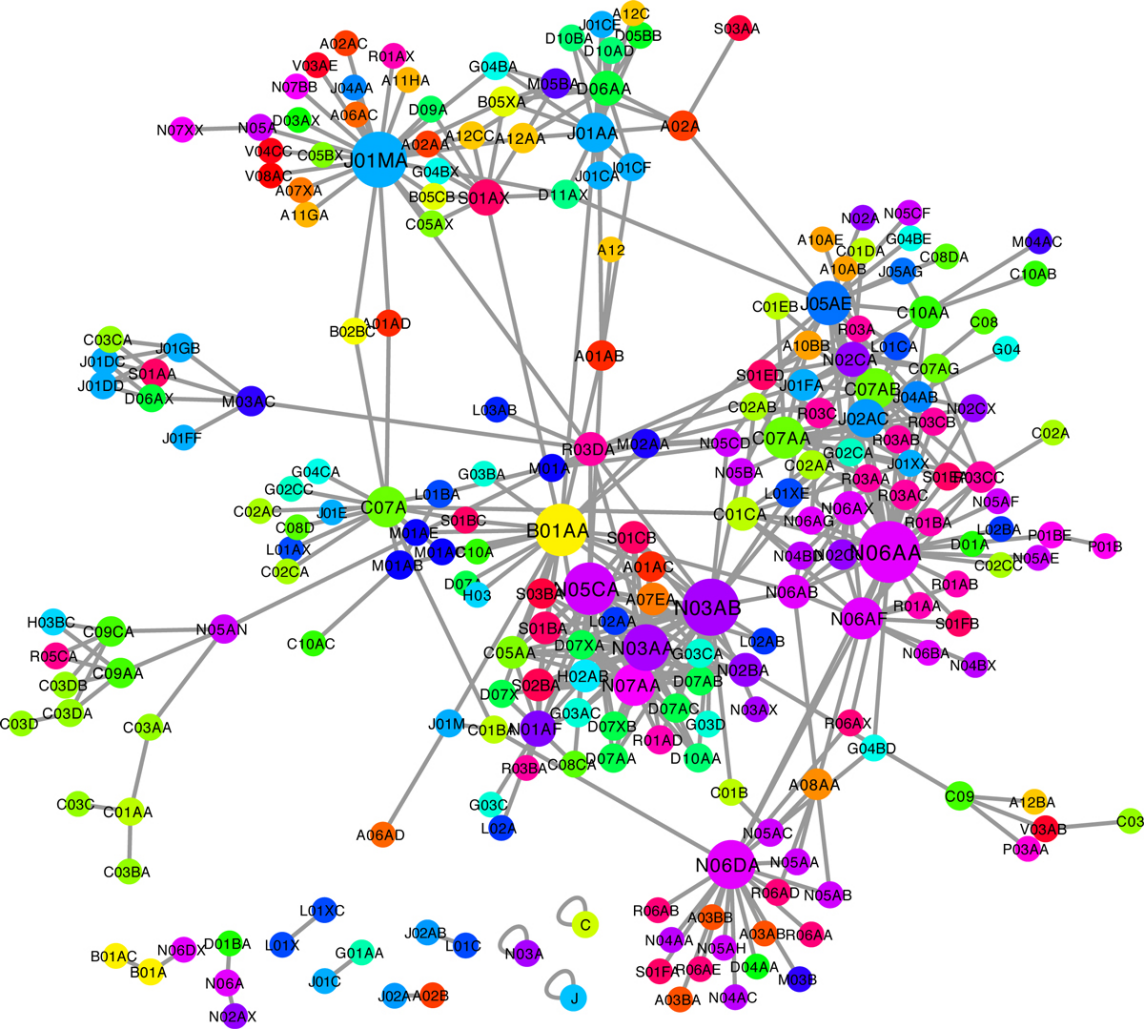
**Systematic DG-DG interaction network**.

To investigate systematic interaction map among functional drug-groups, we constructed DG-DG interaction network which consists of statistically significant DG-DG interactions (Figure 2). We first expected that analysis based on DG-DG interaction could provide systematic, contextual and intuitive knowledge about DDI better than analysis based on only DDI.

The topological analysis of the DG-DG network showed that it had little modular property that the neighbours tended to be disconnected (low clustering coefficient). This kind of network architecture seems to be reasonable in DDI network because the drugs that have a common function usually are not taken together in clinical use. In addition, although most of DGs had a few links in the network, a small number of DGs had a large number of links (hub DGs) and might have their own specific DDI mechanisms (Figure 2 and Table 1). For example, the most highly-linked DG was non-selective monoamine reuptake inhibitors (N06AA), and the drugs in the group had various descriptions on DDI, which were serotonin syndrome in concomitant therapy with other serotonin modulators (N06AB, N02CC, N06AX), increase in the toxicity (J01AA, J05AE), antagonistic effect (N06DA, N02CX), additive QTc prolongation(N05AE, L02BA, P01BE), and so on.

**Table 1:** Highly-linked drug groups

ATC	Summary	Number of links
N06AA	Non-selective monoamine reuptake inhibitors	32
N03AB	Hydantoin derivatives	28
J01MA	Fluoroquinolones	27
N05CA	Barbiturates, plain	24
B01AA	Vitamin K antagonists	24
N06DA	Anticholinesterases	21
N03AA	Barbiturates and derivatives	19
J05AE	Protease inhibitors	17
N07AA	Anticholinesterases	15
N06AF	Monoamine oxidase inhibitors, non-selective	15
C07AA	Beta blocking agents, non-selective	15
C07AB	Beta blocking agents, selective	14
C07A	BETA BLOCKING AGENTS	14
J01AA	Tetracyclines	13
J02AC	Triazole derivatives	12
S01AX	Other antiinfectives	10
N01AF	Barbiturates, plain	10
D06AA	Tetracycline and derivatives	9
N02CA	Ergot alkaloids	9
N06AB	Selective serotonin reuptake inhibitors	8

On the other hand, the drugs in N03AB group which had the second largest degree were mostly related to many different cytochrome P450 mechanism (substrate, inhibitor, or inducer of CYP2C8, CYP2C19, CYP3A4, CYP2B6, CYP1A2, CYP2D6, and CYP2C9), which seems to be a potential common DDI mechanisms of DG N03AB. In addition, the drugs in J01MA such as fluoroquinolone (quinolone antibacterials) had DDIs with calcium, magnesium, zinc, and aluminium by formation of non-absorbable complexes.

The drug groups acting on nervous system such as N06AA, N03AB, N05CA, N06DA, N03AA and N07AA tended to have many DG-DG interactions including as many as thirteen anatomical main groups (A:alimentary tract and metabolism, B:blood and blood forming organs, C:cardiovascular system, D:dermatologicals, G:genito-urinary system and sex hormones, H:systemic hormonal preparations, excluding sex hormones and insulin, J:anti-infectives for systemic use, L:antineoplastic and immune-modulating agents, M:musculo-skeletal system, N:nervous system, P:antiparasitic products, insecticides and repellents, R:respiratory system, S:sensory organs). In addition, group P interacted with C, N, and P; group H with C, N, and B. Lastly, the DGs that had the similar DDI patterns tended to have similar therapeutic effects (same colour in Figure 2), suggesting that DDIs contain the information about drug mechanisms. In addition, other properties of the network are shown in Table 2, and degree distribution of the DG-DG interaction network is shown in Additional file 1.

**Table 2:** Characteristics of DG-DG networks

	DG-DG Network (Figure 2)	Secondary Network (Figure 4)
Clustering coefficient	0.057	0.688
Connected components	10	10
Network diameter	12	3
Network radius	1	1
Network centralization	0.117	0.231
Shortest paths	43910 (85%)	406 (17%)
Characteristic path length	4.303	1.517
Average number of neighbours	3.700	4.583

### Secondary network of DGs sharing similar DG-DG interaction patterns

**Figure 3:**
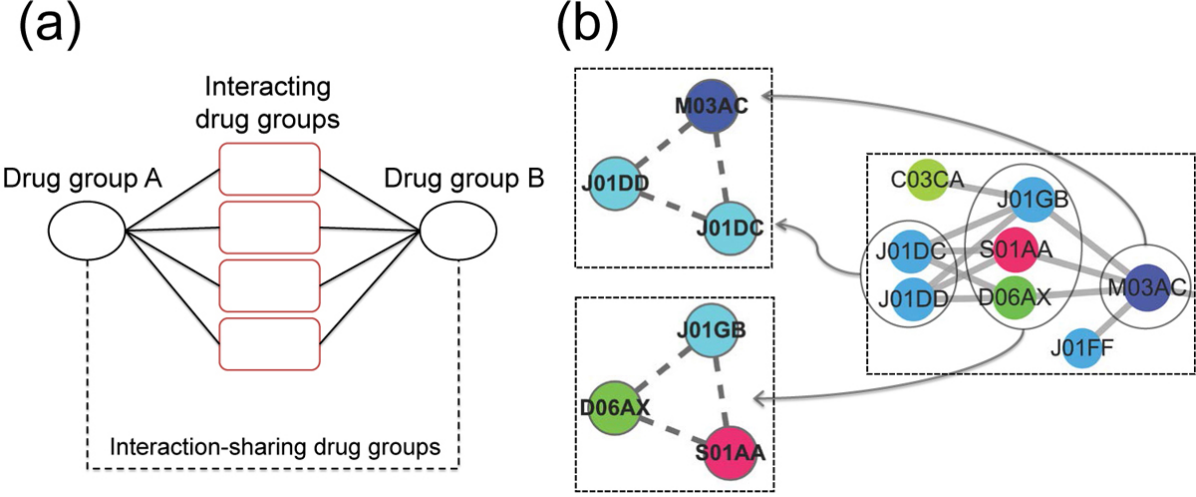
**Schematic diagram representing the construction of secondary network based on interaction sharing ratio**.

**Figure 4:**
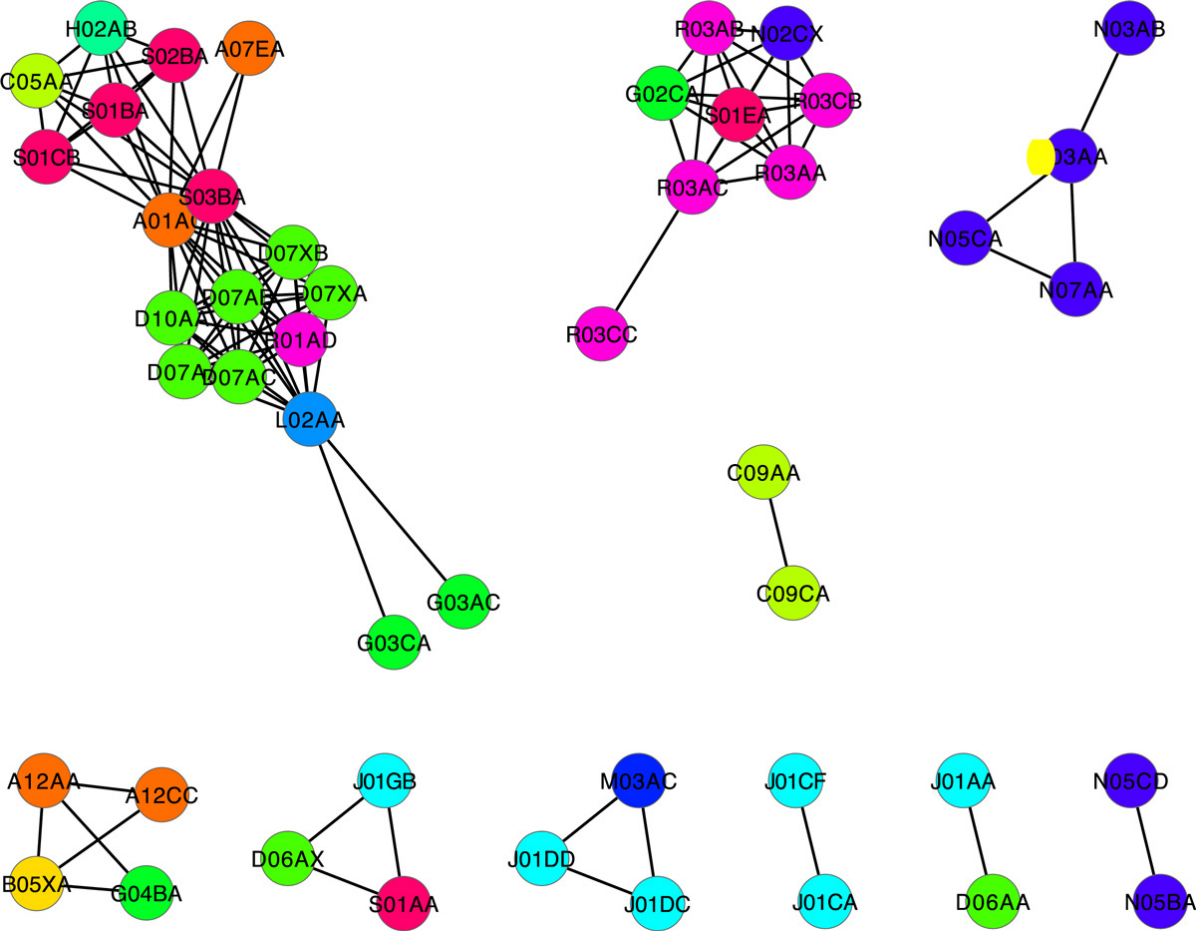
**Secondary DG-DG network based on interaction sharing ratio**.

In the DG-DG interaction network, we found that some DGs were sharing the set of DG-DG interaction partners, which led to the construction of secondary DG-DG network based on DG-DG interaction partner sharing ratio. The procedure assumed that the DGs which had common DG-DG interaction partners could have similar drug mechanisms. To collect this kinds of DGs, we calculated the ratio measuring how many DGs are common partner of particular two DGs (Figure 3a), and applied these ratios to construct the secondary DG network (Figure 3b). After applying two statistical conditions: 1) fraction of common DG-DG interaction partners > 75%, and 2) hyper geometric p-value < 0.01. The resulting DG network is shown in Figure 4. In contrast to the previous network (Figure 2), it exhibited a highly modular topology where the DGs in the modules were densely connected to each other.

### Network modules that consist of DGs sharing drug-interactions

The MCODE method [27] was applied for detecting highly-linked modules, which resulted in seven network modules (Table 3). Because the drug-groups in the same module would have similar drug-interaction patterns, they likely share the common mechanisms for the drug-interactions and/or therapeutic effects.

**Table 3:** Functional modules in drug-interaction sharing network

Cluster	Score (Density × Number of Nodes)	Number of nodes	Number of edges	Members
1	4.5	10	45	D10AA, R01AD, D07XA, D07XB, A01AC, L02AA, D07AB, D07AC, D07AA, S03BA
2	3	7	21	R03AA, N02CX, G02CA, S01EA, R03CB, R03AC, R03AB
3	2	5	10	S02BA, C05AA, S01CB, S01BA, H02AB
4	1.25	4	5	G04BA, A12AA, A12CC, B05XA
5	1	3	3	J01DC, J01DD, M03AC
6	1	3	3	D06AX, J01GB, S01AA
7	1	3	3	N03AA, N07AA, N05CA

The first module in Figure 4 (Cluster 1 in Table 3) contained the largest number of DGs, and, expectedly, most of them had the same therapeutic class which is corticosteroids. Although the majority of the module is composed of D07xx (xx denotes ambiguous ATC subclass), the other ATC classes L02AA (estrogens), A01AC (corticosteroids for local oral treatment), and S03BA (corticosteroids and anti-infectives in combination) also belonged to the similar therapeutic classes. The second module contained seven DGs. Most of them are sympathomimetics drugs that mimic the effects of transmitter substances of the sympathetic nervous system. Similar to the first module, the major DGs are R03xx (drugs for obstructive airway diseases) and the other DGs such as G02CA (sympathomimetics, labour repressants), S01EA (Sympathomimetics in glaucoma therapy), and N02CX (antimigraine preparations) also had the similar drug effects. In addition, their drug-interactions are mainly described by antagonism which can be an evidence of the similar molecular mechanisms of the DGs in this module. The module which consists of A12AA (calcium), A12CC (magnesium), B05XA (electrolyte solution), and G04BA (acidifier) showed the common drug-interaction forming non-absorbable complex, which might be due to the common ionization property.

Among the triangularly-linked modules, N05CA (barbiturates, antiepileptics), N07AA (anticholinesterases), and N03AA (barbiturates and derivatives, psycholeptics) commonly had an effect on nervous system but had different therapeutic subclasses. The other triangular groups, J01GB (other aminoglycoside antibacterials), D06AX (Other antibiotics for topical use), and S01AA (antibiotics, ophthalmologicals) were also commonly the antibiotics in spite of the different target organisms. Interestingly, two betalactam antibacterial drug-groups (J01DD and J01DC) were connected to muscle relaxants (M03AC). By literature search, we found that these two drug groups had the unexpected cross-reactivity.

In summary, the results suggested that information on DG-DG interaction partner sharing can be one useful feature to infer the mechanisms of DDI and therapeutic effects, and also, to reposition the existing drugs for another use.

### DDI contains information on various drug features

From the analysis of the DG-DG network, we hypothesized that the drugs sharing common Drug-DG interaction partners might have similar drug mechanisms. To test this hypothesis, the Drug-DG partner sharing ratio was calculated for every drug pair to investigate common drug features between them. This sharing ratio was calculated by the same way as constructing Figure 4.

Instead of using Drug-Drug interaction, considering Drug-DG interactions in the calculation of the sharing ratio between drugs could have a beneficial effect for the reason that holistic method can compensate the noise caused by almost identical drugs.

**Figure 5:**
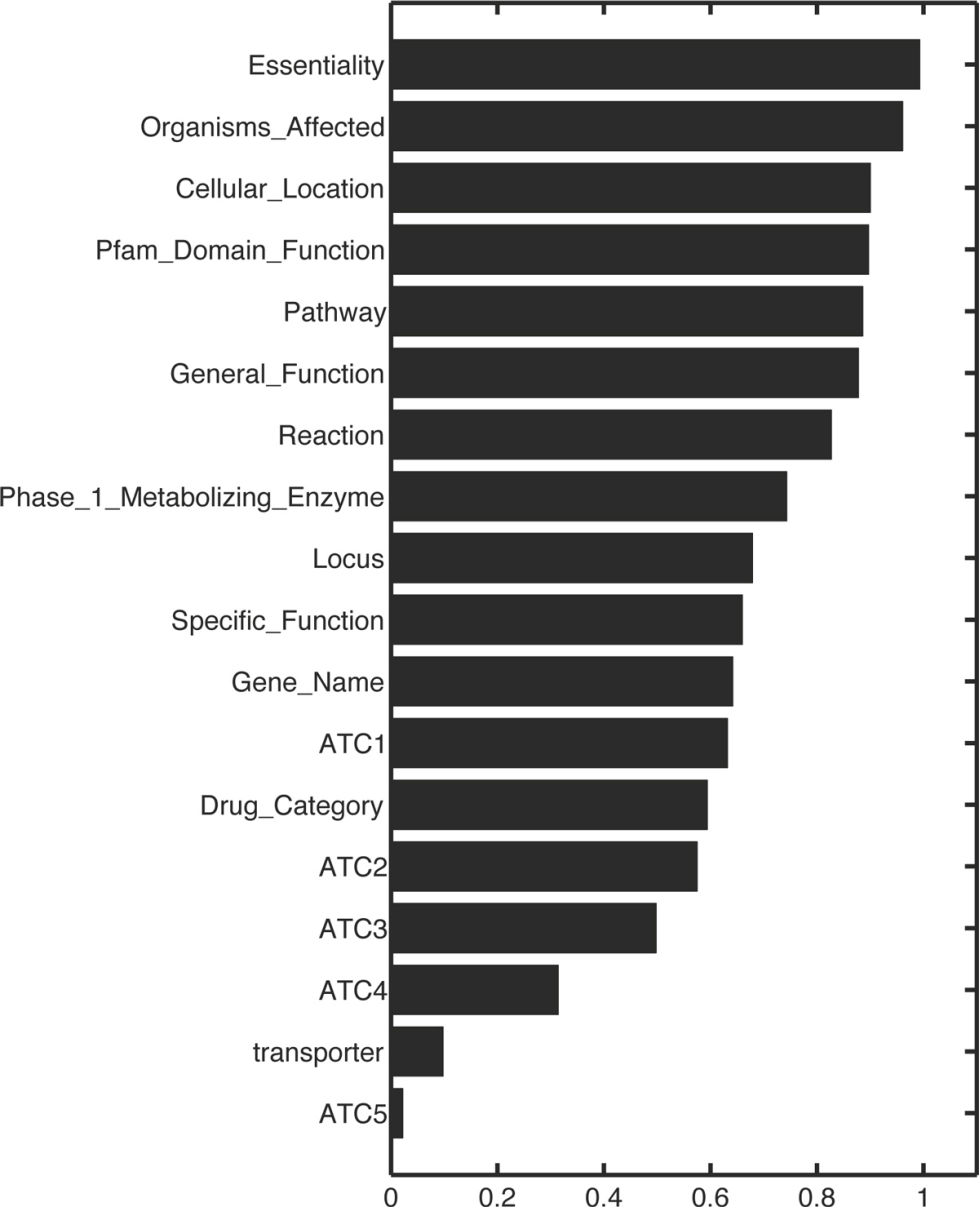
**Marginal feature-matching ratio for the interaction sharing drugs**.

For the drug-pairs having the high ratio, we checked whether the pair of drugs had the same features and the results are shown in Figure 5, Additional file 2 (drug's feature), and Additional file 3 (target's feature). The results showed that the drug-pairs sharing Drug-DG interaction partners have the common target-related features such as 'target cellular location', 'target pathway', and 'target domain function', and common drug-related features such as' general drug function and phage 1 metabolizing enzyme'.

In addition, features of each drug (e.g. prediction of drug target) were predicted by those of interaction-sharing drug (ISD) based on our hypothesis that an enriched common feature of ISDs is also likely to be a feature of the query drug. After collecting all ISDs, the features of the collected drugs were scored by frequency.

Simply, when the most frequent common feature for each drug was assigned to the feature of query drug, those of 80% were correctly predicted.

### Drug interaction-sharing but dissimilar drugs

**Figure 6:**
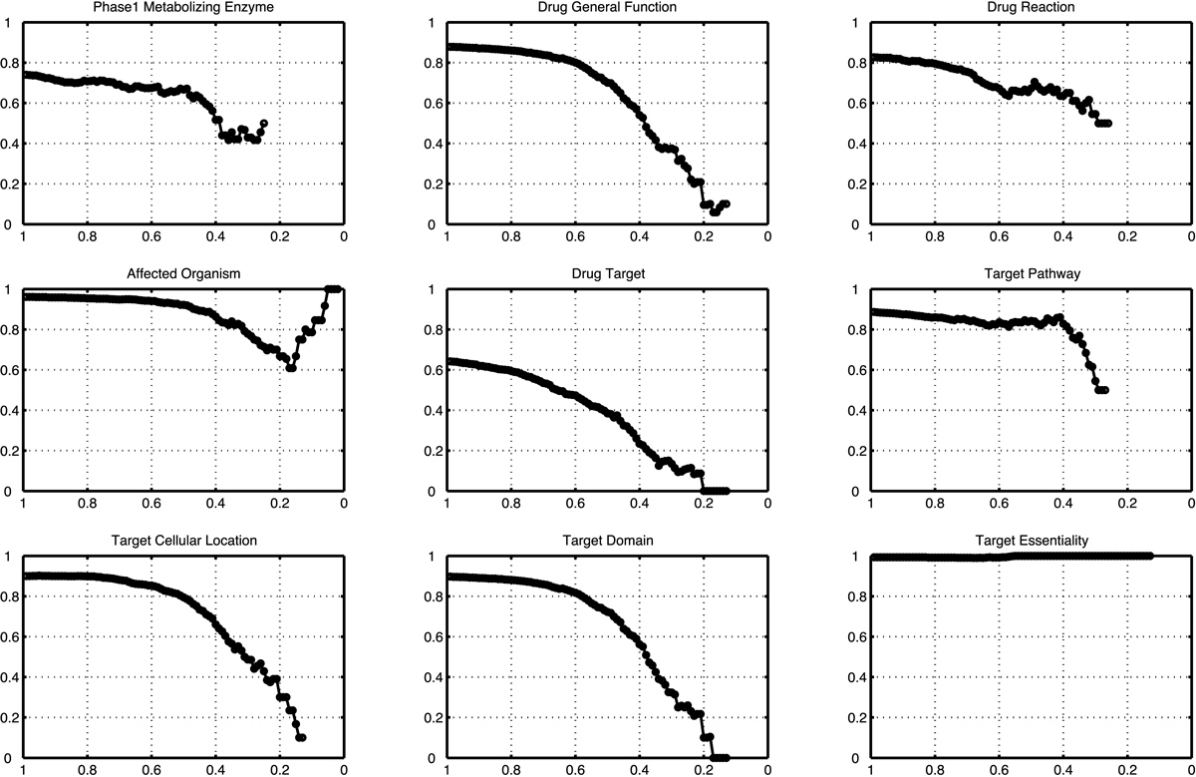
**Drug feature-matching ratio according to the different structural similarity cut-offs**.

More interesting study on Drug-DG interaction would be whether it contained unique information compared to the typical structural similarity. Do the structurally dissimilar but ISDs have any common drug features? If there are these features, what kinds of drug features are uniquely represented by our interaction information? To answer the questions, we investigated various types of drug features and structural similarity among chemical structures of drugs (Figure 6).

The relationship between chemical structure similarity and the feature-matching ratio for the interaction-sharing drugs was analysed with nine different drug features in Figure 5. Figures for all drug features were shown in Additional file 1 and Additional file 2. In Figure 6, feature-matching ratios (vertical axis) were plotted along the threshold of structural similarity (horizontal axis). For example, 0.5 in X-axis means that only the drug pairs whose structural similarity is less than 0.5 are only considered to calculate feature-matching ratios. 0.5 in Y-axis means that 50% of pairs share the same value of corresponding feature. It has to be noted that only the drugs which had the same type of annotations were considered for calculating the matching-ratio. The results showed that feature-matching ratio for most drug features were decreased as structurally dissimilar drugs were considered. Obviously 'phage1 metabolizing enzymes' seemed to be an important common factor for sharing drug-interactions regardless of structure similarity. However, interestingly, some drug features (e.g. 'general drug function', 'target reaction', and 'target pathway') maintained high matching ratio higher than 60% even when the structure similarity was around 0.4, which suggested that sharing drug-interactions contain not only information of pharmacodynamics such as 'target pathway' and 'target domain function', but also pharmacokinetic information such as metabolizing enzyme and transporter. In particular, 'target pathway' information showed greater than 80% matching ratio when the structure similarity was even ambiguous (0.4~0.5) while 'drug target' identity was very low.

On the other hand, the 'target essentiality' showed almost 100% matching ratio independent on structural similarity. Taken all together, we can conclude that the information on drug-drug interaction could be another unique source to describe various drug mechanisms with other chemical similarity measures.

### Are DG-DG networks more useful than drug-drug interaction network?

In this work, we have showed that a DG-DG interaction network, its supplemental network and neighbour sharing information are intuitive and useful. This may misleads people thinking DG-DG interaction network gives more accurate information than DDI network does. Grouping drugs is a kind of abstraction, and the main purpose of our work is to show the concept of DG and to provide intuitive information. Thus, we expect complementary use of both DG-DG and drug-drug interactions will show more accurate and sensitive inference.

We used one ATC classification to bundle drugs, but this classification is not a unique method to categorize drugs. Our main concept can be applied with any kinds of drug classification (e.g. chemical type). More sophisticated way to define Drug Group will be able to improve DG-DG network and to provide novel kinds of information. We suggest that constructing DG-DG networks with different definitions of DG is new way to view novel dimension of drug interaction. Further studies are welcomed to validate.

## Conclusion

In this study, we carried out systematic analysis on functional DG-DG interactions which provide more contextual and intuitive view about DDI. From the interaction map, we also constructed the secondary network which consists of DGs sharing drug interactions. The detected modules of the network represent the similar functional DGs in spite of different annotation of therapeutic class, suggesting that drug interactions contain the information about mechanisms of drugs. In addition the usefulness of our work in drug repositioning was shown with the example of betalactam antibacterial drugs and muscle relaxants. Moreover, we questioned whether DG-DG interaction information can be used to infer drug's mechanisms. The results show that some drug features such as metabolizing enzyme, drug function, and target pathway are well described by interactions even for the case that two drugs have low structural similarity. Thus, we expect that information on DG-DG interaction can be utilized as a novel useful source to describe drug's mechanisms.

## Methods

### Collection of drug-drug interaction and drug features

Descriptions on drug-drug interaction were retrieved from 'Drug_Interactions' records in DrugBank database [28]. To define DDI pairs from the information, various drug names were unified by mapping synonyms, brand names generic name of each drug to a unique DrugBank ID. The descriptions that could not be mapped by DrugBank ID were discarded. As a result, 10,759 DDIs of 1,075 drugs were detected. In addition, various features of drugs and drug targets such as phage1 metabolizing enzyme, transporter, specific/general drug functions, drug category, ATC codes, drug reaction, affected organism; target information: drug target, pathway, cellular location, locus, domain function, and essentiality were also collected with the drugs.

### Functional grouping of drugs by ATC code

Functional grouping of drugs was performed by mapping ATC codes into drugs. Anatomical Therapeutic Chemical (ATC) Classification System divides drugs into 5 different levels according to the organ or system on which they act and/or their therapeutic and chemical characteristics. One drug can have more than one code. Based on the drug classification system and its hierarchical structure, functional drug groups were defined, thus, each DG corresponds to each ATC code.

### Statistical significance of DG-DG interactions

To calculate the statistical significance of the number of drug interactions between two ATC groups, ten thousands of random pairs of drug groups were generated for each unique combination of DG pairs and we counted how many drug interactions exists in the each random pair. Then p-value was calculated based on these distributions.

### Interaction network between functional DGs of various levels

Significant DG-DG interactions (p-value < 0.0001) were considered to construct DG-DG interaction network. Note that ATC codes in different levels could be defined as a significant interaction because we tested all ATC codes regardless of the hierarchical structure. In addition, due to this hierarchical structure, some group interactions seemed to be redundant. For example, even though C01AA group was linked to both of C03BA and C03B, the interaction between C01AA and C03B just stemmed from more specific interaction between C01AA and C03BA. All of the redundant interactions were removed in constructing DG interaction map.

### Interaction partner sharing ratio between DGs and between drugs

The interaction partner sharing ratio, how much interaction profile is similar, was measured between drug-groups or drugs. Specifically, drug groups (or drugs) were represented by the set of their interactive ATC groups (i.e. bit string). Based on the representation, all possible DG pairs (or drug pairs) were compared for measuring how much they are sharing the interactive ATC groups. The similarity measures used in this procedure were hyper geometric p-value measuring overlapping significance (p-value < 0.01) and Tanimoto coefficient (ration of common bits to union bits > 0.75). The DG pairs or drug pairs satisfying the two criteria were used to construct the secondary DG network based on the tendency sharing drug-interaction profiles.

### Network module detection

Molecular Complex Detection (MCODE) was applied to identify densely connected modules in the secondary DG-DG network based on partner-sharing ratio. The method uses graph-theoretic clustering based on vertex weighting by local neighbourhood density and outward traversal from a locally dense seed node. Module score of each cluster was defined by the ratio of the number of edges to the number of nodes in each cluster.

### Calculation of chemical structure similarity

Chemical structure similarity between drugs was based on 881-bit PubChem [29] fingerprints calculated by PaDEL software [30]. Specifically, the fingerprint was calculated for each drug, and Tanimoto coefficient (ratio of intersection-bits to union-bits) was used as a 'chemical structural similarity' in this study.

## Competing interests

The authors declare that they have no competing interests.

## Authors' contributions

ML and KP conceived and implemented the concepts and methods. ML and KP wrote the manuscript, DK supervised the work. All authors read and approved the final manuscript.

## Supplementary Material

Additional file 1 Degree distribution of drug-group interaction network Click here for file

Additional file 2 Drug-related feature-matching ratio Click here for file

Additional file 3 Target-related feature-matching ratio Click here for file

## Declarations

The publication costs for this article were funded by a grant of the Korean Health Technology R&D Project, Ministry of Health & Welfare, Republic of Korea (A120017).

This article has been published as part of *BMC Systems Biology *Volume 7 Supplement 3, 2013: Twelfth International Conference on Bioinformatics (InCoB2013): Systems Biology. The full contents of the supplement are available online at http://www.biomedcentral.com/bmcsystbiol/supplements/7/S3.
