# Supplementary figures and images for: Interaction network among functional drug groups
Source: BMC Syst Biol. 2013 Oct 16;7(Suppl 3):S4. doi: 10.1186/1752-0509-7-S3-S4 (PMC3852121; doi:10.1186/1752-0509-7-S3-S4)

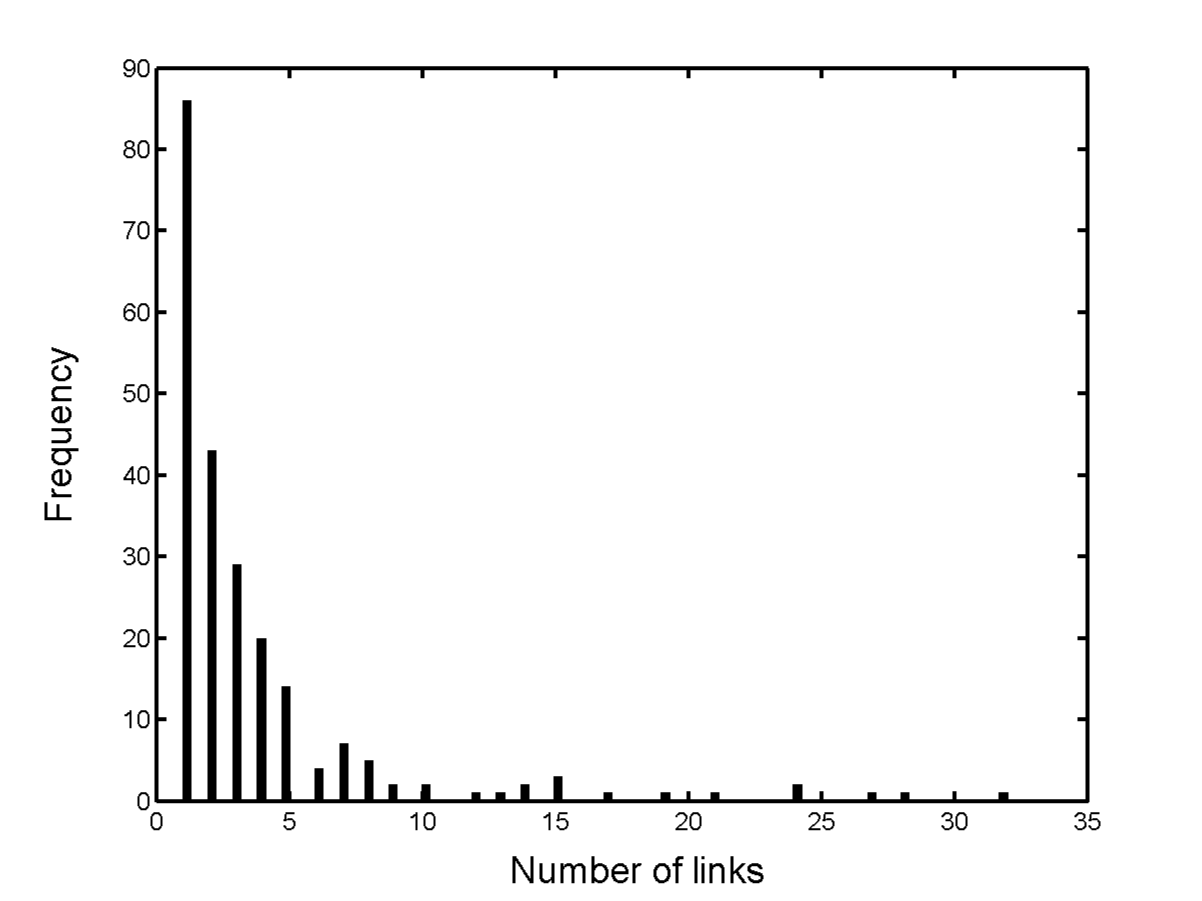

Supplement: Additional file 1 — Degree distribution of drug-group interaction network [file 1752-0509-7-S3-S4-S1.png]

Drug Target

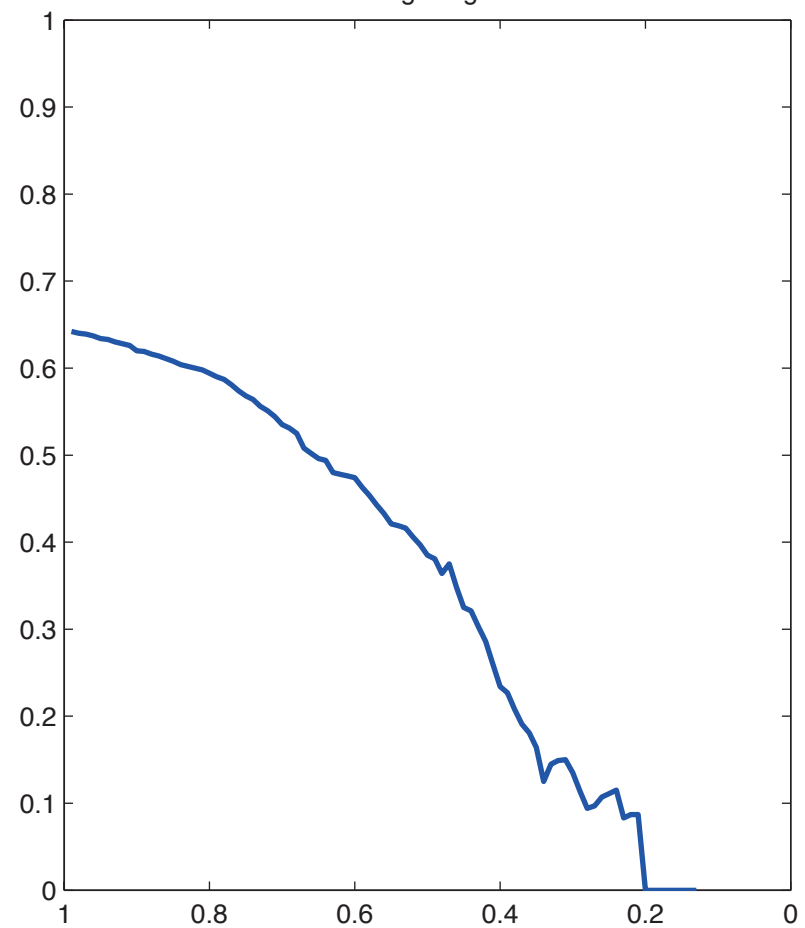

Pathway

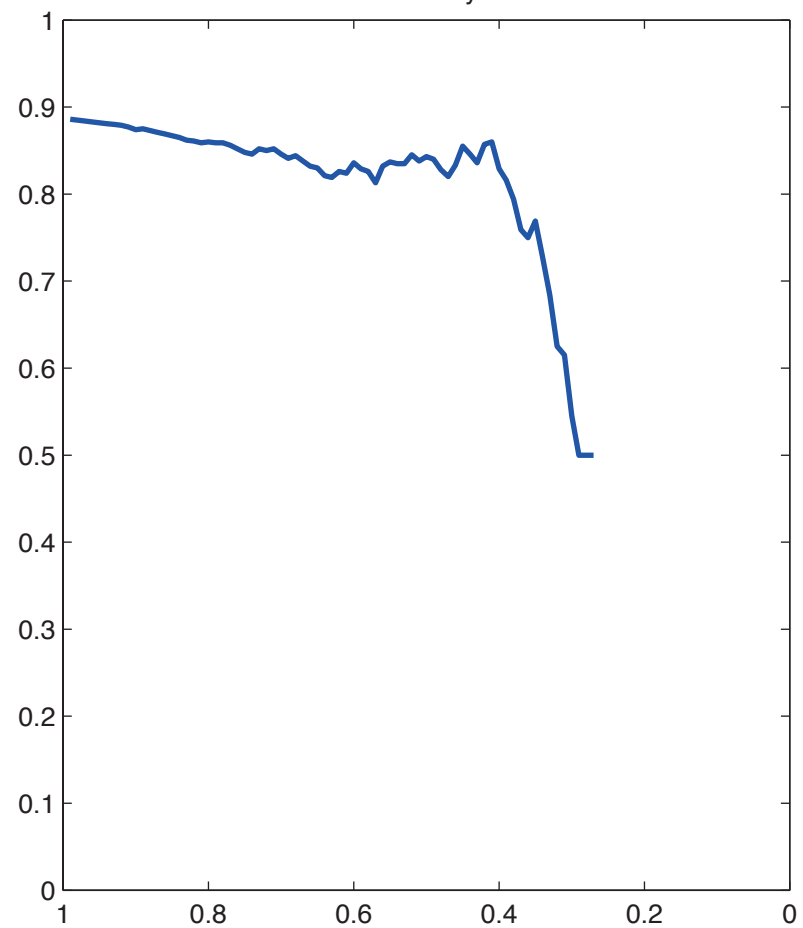

Cellular Location

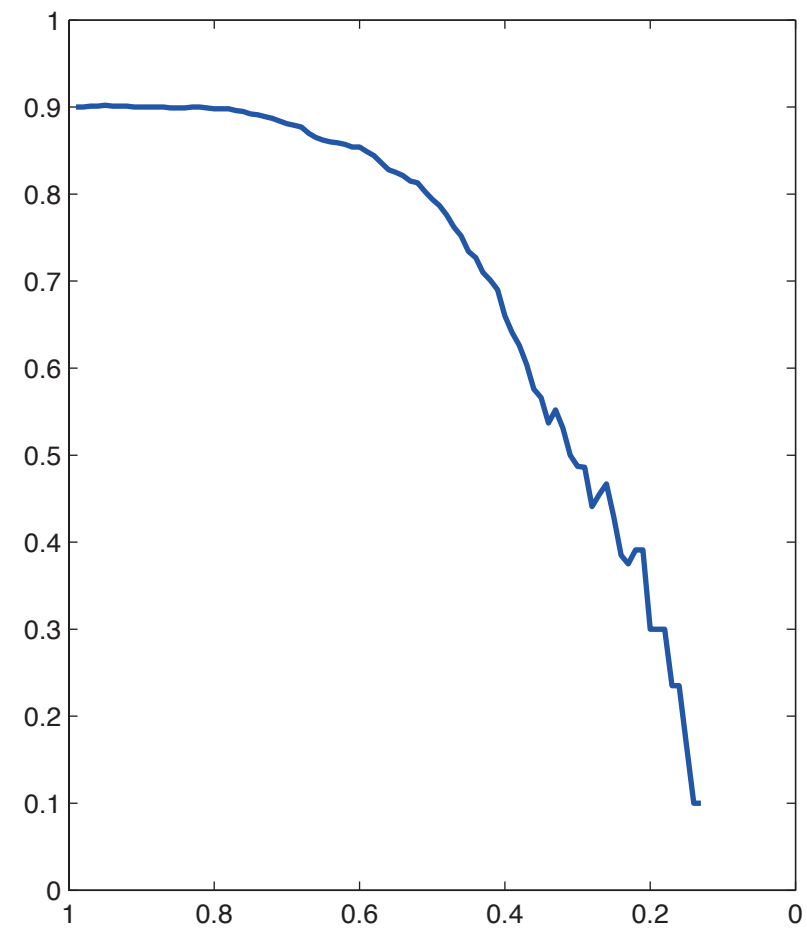

Locus

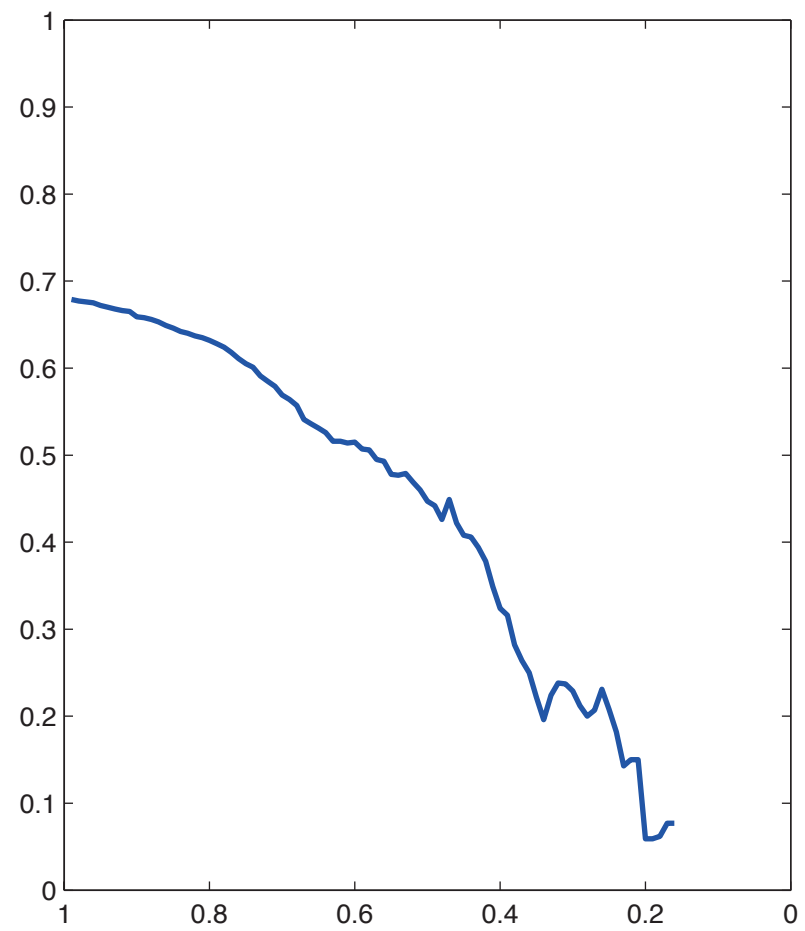

Domain Function

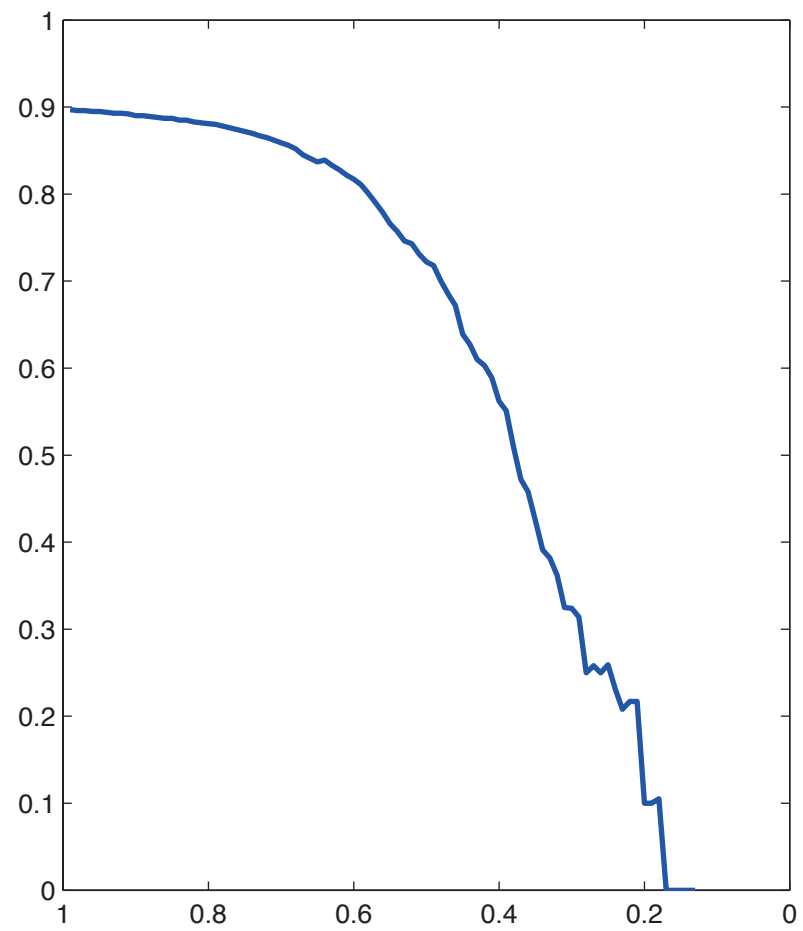

Essentiality

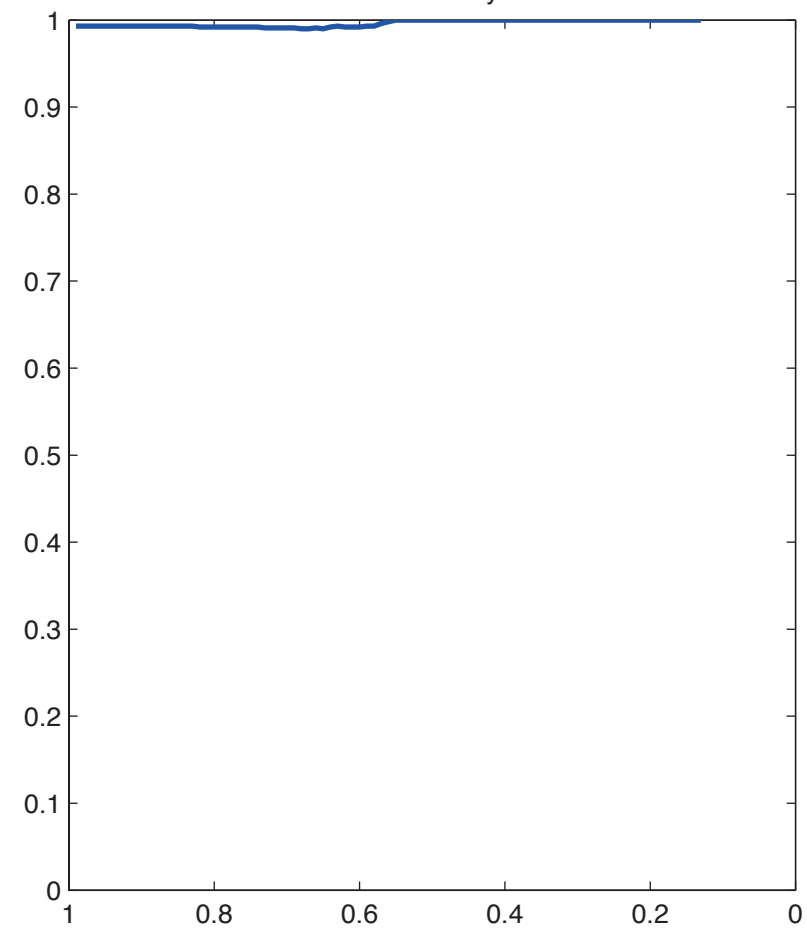

Supplement: Additional file 3 — Target-related feature-matching ratio [file 1752-0509-7-S3-S4-S3.pdf]
