# Supplementary material for: Interaction network among functional drug groups
Source: BMC Syst Biol. 2013 Oct 16;7(Suppl 3):S4. doi: 10.1186/1752-0509-7-S3-S4 (PMC3852121; doi:10.1186/1752-0509-7-S3-S4)
Supplement: Additional file 2 — Drug-related feature-matching ratio [file 1752-0509-7-S3-S4-S2.pdf]

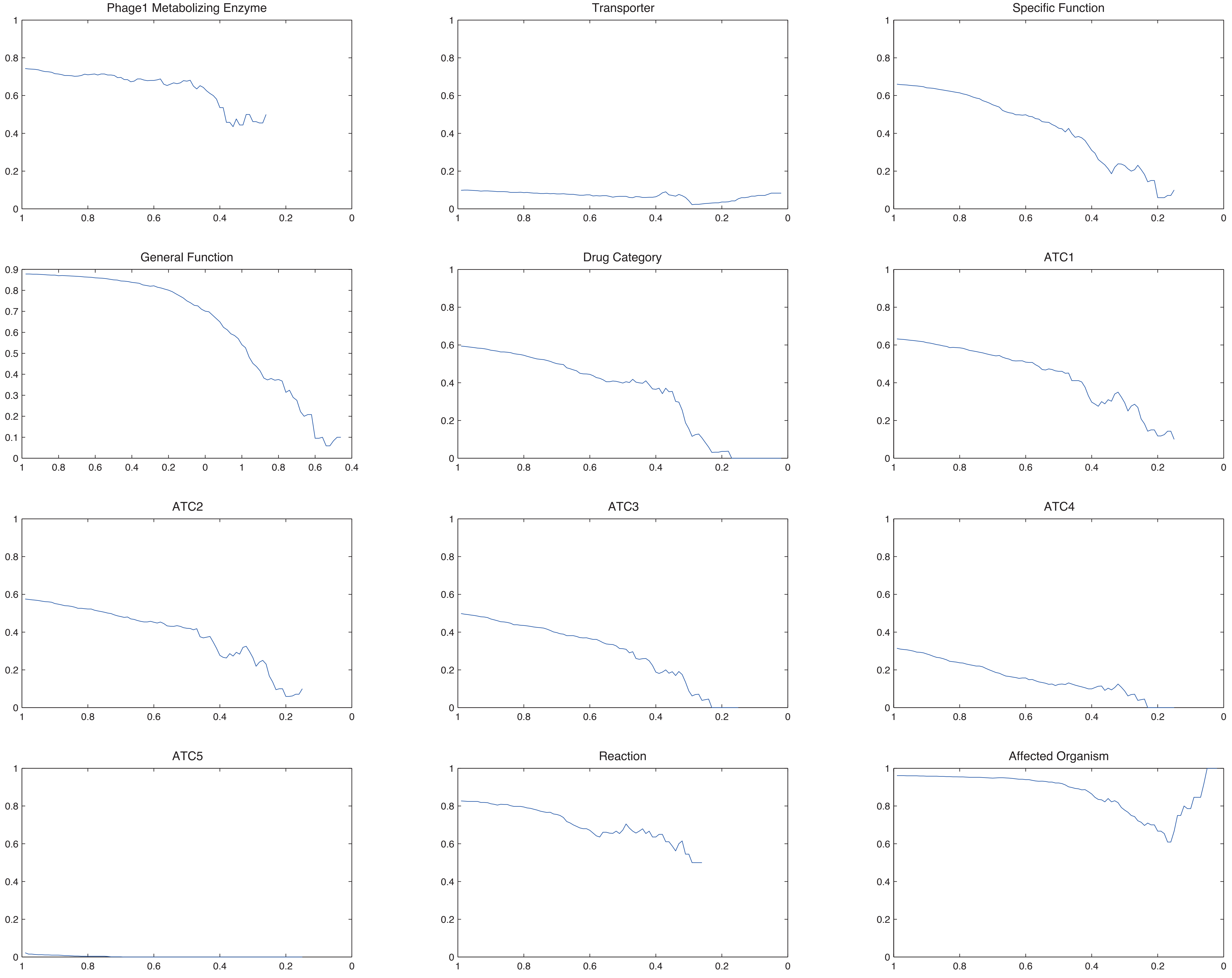
Phage1 Metabolizing Enzyme
Transporter
Specific Function
General Function
Drug Category
ATC1
ATC2
ATC3
ATC4
ATC5
Reaction
Affected Organism
0 0.2 0.4 0.6 0.8 1
0 0.1 0.2 0.3 0.4 0.5 0.6 0.7 0.8 0.9
1 0.8 0.6 0.4 0.2 0
1 0.8 0.6 0.4 0.2 0 1 0.8 0.6 0.4
